# Draft Genome Sequence of the Clinical Isolate *Acinetobacter nosocomialis* Strain M2

**DOI:** 10.1128/genomeA.00906-13

**Published:** 2013-11-07

**Authors:** Michael D. Carruthers, Christian M. Harding, Beth D. Baker, Robert A. Bonomo, Kristine M. Hujer, Philip N. Rather, Robert S. Munson

**Affiliations:** Center for Microbial Pathogenesis, the Research Institute at Nationwide Children’s Hospital and Department of Pediatrics, The Ohio State University College of Medicine, Columbus, Ohio, USAa; Research Service, Louis Stokes Cleveland Veterans Affairs Medical Center, Cleveland, Ohio, USAb; Research Service Veterans Affairs Medical Center, Decatur, Georgia, USA, and Department of Microbiology and Immunology, Emory University School of Medicine, Atlanta, Georgia, USAc

## Abstract

We report the 3.78-Mbp high-quality draft assembly of the genome from a clinical isolate of *Acinetobacter nosocomialis* called strain M2 (previously known as *Acinetobacter baumannii* strain M2).

## GENOME ANNOUNCEMENT

*Acinetobacter nosocomialis* is a member of the *Acinetobacter calcoaceticus-Acinetobacter baumannii* (ACB) complex, which is composed of *A. calcoaceticus, A. baumannii, Acinetobacter pittii* (formerly genomic species 3), and *A. nosocomialis* (formerly genomic species 13 TU) (1). Except for *A. calcoaceticus*, members of the ACB complex are opportunistic pathogens that pose a significant threat to human health through their ability to cause severe infections. This threat is compounded by both the high incidence of ACB complex infections in critically ill patients and the prevalence of antibiotic resistance exhibited by members of the ACB complex (2).

*A. nosocomialis* strain M2, isolated in 1996 from a hip infection of a patient at Cleveland MetroHealth Systems (Cleveland, OH), has been studied in several laboratories. An acyl-homoserine lactone autoinducer synthase from strain M2 has been isolated and characterized (3). Nonnative acyl-homoserine lactones have been shown to attenuate strain M2 quorum sensing (4). A novel mechanism of fluoroquinolone resistance has been discovered through the mutagenesis of strain M2 (5). The twitching motility exhibited by strain M2 is mediated by a type IV pilus system, which also mediates the natural transformation phenotype of strain M2 (6). Strain M2 is also motile on the surface of agar plates through an unknown (7) Tfp-independent mechanism (6). In addition, strain M2 is able to outcompete other bacteria using a type VI secretion system (8).

Genomic DNA was prepared using the Qiagen DNA purification kit. Genome sequencing was performed using the Illumina HiSeq and MiSeq platforms maintained at the Research Institute at Nationwide Children’s Hospital Biomedical Genomics Core (http://genomics.nchresearch.org). A total of 4 million paired-end MiSeq reads (300-bp insert size) trimmed to 200 bp and 4 million mate-pair HiSeq reads (3-kb insert size) trimmed to 35 bp were assembled *de novo* using SeqMan NGen (version 4.1.2; DNAStar, Madison, WI). Postassembly manipulation was performed using SeqMan Pro (DNAStar). This genome assembly yielded 11 contigs comprising a total genome length of 3,782,411 bp and an N_50_ of 556 kb, with 163-fold average coverage. Genome annotation using the NCBI Prokaryotic Genomes Automatic Annotation Pipeline (version 2.0) predicted a total of 3,487 open reading frames.

Previously, strain M2 was classified by multilocus sequence typing (MLST) as belonging to the *A. baumannii* species (R. A. Bonomo, unpublished data). Two currently available MLST schemes were used to retype strain M2. Four of the seven genes required for MLST using the “Oxford” scheme (see http://pubmlst.org) were identified in the M2 genome. Based upon this analysis, strain M2 is most closely related to an *Acinetobacter* sp. 13TU isolate from Brazil. Five of the eight genes required for MLST using the “Pasteur” scheme (see http://www.pasteur.fr/mlst) were identified in the strain M2 genome. An analysis of these alleles indicates that strain M2 is most closely related to *Acinetobacter* sp. 13TU strain RUH 2210. Based on this evidence, we recommend that *A. baumannii* strain M2 be referred to as *A. nosocomialis* strain M2.

### Nucleotide sequence accession numbers.

This whole-genome shotgun project has been deposited at DDBJ/EMBL/GenBank under the accession no. AWOW00000000. The version described in this paper is version AWOW01000000.
